# A workplace well-being game intervention for health sciences librarians to address burnout

**DOI:** 10.5195/jmla.2020.742

**Published:** 2020-10-01

**Authors:** Tallie Casucci, Amy B. Locke, Autumn Henson, Fares Qeadan

**Affiliations:** 1 tallie.casucci@utah.edu, J. Willard Marriott Library, University of Utah, Salt Lake City, UT; 2 amy.locke@hsc.utah.edu, Family and Preventive Medicine, University of Utah, Salt Lake City, UT; 3 autumn.henson@utah.edu, Public Health, University of Utah, Salt Lake City, UT; 4 fares.qeadan@utah.edu, School of Medicine, University of Utah, Salt Lake City, UT

## Abstract

**Objective:**

The authors measured burnout among health sciences librarians at their institution and determined whether a serious game intervention could improve personal and workplace well-being.

**Methods:**

A modified American Medical Association Mini-Z burnout survey was administered to library faculty in 2016 and both library faculty and staff in 2017. A three-month team-based game was implemented and assessed as an intervention to improve well-being among library employees. After the game, the burnout survey was re-administered to employees in 2018.

**Results:**

Library faculty scored poorly on burnout indicators, with 38%–73% of faculty reporting emotional exhaustion and 54%–91% reporting job-related stress over the years. In 2017, 62% of library staff members reported experiencing burnout and 38% indicated they felt a great deal of stress because of their jobs. Regarding the game intervention, 70% of post-game survey respondents reported that the game encouraged them to socialize with colleagues. Qualitative coding of survey responses resulted in 4 themes describing the most enjoyable aspects of the game: sociability, motivation, game play, and fun. Employees found that the game was a useful strategy for encouraging a more social culture with fun activities.

**Conclusions:**

Similar to previous studies of librarians and health professionals, health sciences librarians at our institution experienced burnout. Although the game intervention did not significantly reduce burnout or increase job satisfaction, it improved collegiality and recognition. Therefore, a workplace well-being game can encourage team building but may not sufficiently address the root causes of health sciences librarian burnout.

## INTRODUCTION

Burnout is defined as a “psychological syndrome of emotional exhaustion, depersonalization, and reduced personal accomplishment” [1]. Emotional exhaustion includes feelings of exhaustion and/or discouragement, which prompts actions to distance oneself emotionally and cognitively from one's work [2]. Depersonalization, or cynicism, is “negative, cynical attitudes and feelings about one's clients” [1], which also leads to distancing and considering others (e.g., colleagues and library users) as impersonal objects. The last element of burnout, reduced personal accomplishment or inefficacy, “refers to the tendency to evaluate oneself negatively…workers may feel unhappy about themselves and dissatisfied with their accomplishments on the job” [1].

These three psychological phenomena happen both in parallel and sequentially under various circumstances. As Maslach writes, “the lack of efficacy seems to arise more clearly from a lack of relevant resources, whereas exhaustion and cynicism emerge from the presence of work overload and social conflict” [2]. To measure the frequency and intensity of burnout, researchers have developed and validated multiple surveys to self-assess burnout risk, including the American Medical Association's (AMA's) Mini-Z survey [3, 4].

If burnout is one end of the continuum, the other end has been described by some authors as optimal workplace well-being or professional fulfillment [2, 5]. Indeed, the World Health Organization defines burnout as “a syndrome conceptualized as resulting from chronic workplace stress that has not been successfully managed” [6] and mental health as “a state of well-being in which every individual realizes his or her own potential, can cope with the normal stresses of life, can work productively and fruitfully, and is able to make a contribution to her or his community” [7]. To Maslach, the antithesis of burnout is engagement [2]. Therefore, when considering burnout, it can be helpful to conceptualize its opposite state, for which Maslach and colleagues describe the three key components of vigor, dedication, and absorption [2].

In the library and information science (LIS) field, many studies have utilized a validated burnout survey tool [8–18], and chapter 2 of Ray's dissertation contains a comprehensive review of LIS burnout literature [12]. Overall, these studies report low to moderate levels of burnout among librarians [8–18]. Burnout in the profession of health sciences librarians has not been investigated, although burnout among systematic review librarians, who likely were mostly health sciences librarians based on the study's recruitment methods, was recently assessed [18].

Burnout prevention interventions range from organization-level strategies to developing personal resilience among employees. The work by Maslach and colleagues on organizational-level predictors of workplace burnout provides a framework for creating organizational-level interventions [2], but these can be extremely challenging to implement. Data support both organization-directed and individual-focused approaches in health care settings [19]. Maslow's hierarchy of needs has also been used to consider target areas [20]. In the LIS field, burnout prevention interventions have not been created or assessed, although McCormack's 2013 book presents ideas for managing burnout at an organizational level in LIS settings [21]. Personal stories from LIS professionals present individualized approaches, such as saying “no,” mediating, and changing jobs [22–26].

Serious games are unique interventions because they address purposes beyond mere entertainment, such as teaching or encouraging new skills, knowledge, or behaviors [27, 28]. Several review articles have shown that, overall, games have a positive impact on participants' engagement [29], knowledge acquisition [30, 31], behavior [31], and motivation [31, 32] and make “health activities fun, enjoyable, and understandable” [32]. For example, serious games have been used in corporate settings for skill or knowledge acquisition or health gamification [33, 34]. Games have not been used in the LIS field for employee interventions but have been discussed as materials for collection development and tools for education and outreach [35–39]. In the context of health sciences libraries, games are mainly for educating students [40–42].

The purpose of this study was to investigate health sciences librarians' burnout before and after a game intervention that was designed to decrease burnout and increase a sense of community.

## METHODS

The University of Utah Resiliency Center conducts annual surveys to address burnout for Spencer S. Eccles Health Sciences Library (Eccles Library) employees. Eligibility to participate in the survey expanded from library faculty members in 2016 to both library faculty and staff in 2017. The Resiliency Center has collaborated with existing programs to implement targeted activities for health sciences librarians during this time frame. An Eccles Library faculty member was charged to develop and assess an intervention between the baseline and follow-up survey. As the Eccles Library had a history of utilizing, creating, and supporting serious games, the authors hypothesized that employees would be receptive to a serious game intervention.

### Survey instrument

The survey instrument included select items from the AMA Mini-Z survey to assess burnout [3, 4]. Eccles Library employees received the survey by email via SurveyMonkey. The survey included 7 out of 10 items originally on the AMA Mini-Z, including 5-point Likert scale questions and a single-item burnout question, validated against the Maslach Burnout Inventory [43]. Categorical cut-offs were used to represent a binary measure of an employee's state, such as job satisfaction (“yes” or “no”), and their symptoms of burnout (“yes” or “no”).

A baseline survey was administered to fifteen library faculty in May 2016 to June 2016 (supplemental Appendix A). A follow-up survey was administered to fourteen library faculty and thirty-nine library staff in September to October 2017 (supplemental Appendix B). Due to extremely high burnout rates found among library employees in 2017, a shorter survey was re-administered to library employees in February 2018 (supplemental Appendix C). This second follow-up survey included questions from the modified AMA Mini-Z burnout survey, questions on depersonalization and meaning in work, and a depression screening (Patient Health Questionnaire-2). Additionally, four free-text response questions addressed next steps for the library moving forward. The raw data were shared with all library employees via email.

Afterward, the Resiliency Center's experts presented at a library employee meeting about available resources, facilitated group meetings on communication and team building, provided individual well-being and resiliency consultations, and hosted regular discussions with library leadership. Also, the Resiliency Center led discussions between Human Resources and library leadership, which resulted in an embedded human resources model.

### Statistical analysis of survey data

The statistical package SAS 9.4 (SAS Institute, Cary, NC, USA) was used to perform statistical analyses. Demographic and AMA Mini-Z items are categorical variables and are reported as frequencies and relative frequencies (column percentages). Fisher's exact tests were used for demographic comparisons because the expected cell frequencies were low due to the small sample size, and Barnard's exact tests for risk difference were used to compare changes in the proportion of “yes” responses to items between years or between faculty and staff within the same year. Statistical significance was defined as *p*0.05. Due to the small sample size and, hence, lack of statistical significance of comparisons, Cohen's h is reported as a measure of effect size, with h=0.20, 0.50, and 0.80 indicating small, medium, and large sized effects, respectively.

### Game design and implementation

After reviewing the baseline survey results and conducting informal interviews with a few library employees, we designed a game intervention, known as the “Wellness Game.” Design box methodology [44] was used to design the game to target personal resilience and develop a culture of well-being. Additionally, the game design gave employees control over their participation, because control over their work and work environment were reported as low in the baseline survey results. Several decisions about game design were made to accomplish these goals (supplemental Appendix D).

After investigating the seven dimensions of wellness [45], we selected four areas for game points that touched on personal health and team collegiality: physical, mental (combined spiritual and intellectual wellness), social, and appreciation. Although appreciation is not a dimension of wellness [45], we recognized that some library employees only heard complaints and problems instead of a happy “Thanks!” from a patron, and a lack of appreciation was noted during the informal interviews with library employees.

During the informal interviews, we also found that library employees missed the “family feel” of the library and opportunities to get to know their colleagues. The game was designed to place a significant focus on this social category through diverse teams and physical game boards. The teams consisted of employees who worked in different departments and physical spaces. The final key game design component allowed flexibility; employees personalized the game with their own goals and interpretations of well-being activities. This decision was strongly supported by library administration, who did not want to further burden employees with additional work or projects. The metrics for determining the success of the game were participation, follow-up survey data, and a sense of community.

Prior to the start of the game, six team captains, who were charged with encouraging their teams, either volunteered or accepted the role when asked. After team captains were identified, all library employees were assigned to six teams. Employees received points for their teams for any activity related to appreciation, social, mental, or physical wellness. Each activity was worth one point, but some activities could receive a bonus point if the player did the activity with a colleague. For example, if someone went on a walk with a colleague, they could receive two points, one for physical wellness and another for social wellness.

Each team had a game board that consisted of a large piece of paper divided into the four categories. All six game boards were attached to a bulletin board that was located in the employee break room to encourage interactions. Employees added tally marks to their teams' game boards in the appropriate category. Additionally, employees nominated others for an award, either serious or funny, through a short online survey. After nominating a colleague for an award, the employee was encouraged to give their team an appreciation point. A link to the survey and a reminder to participate in the Wellness Game were included in the library's daily email. At the game's conclusion, library employees celebrated with a potluck lunch and awards ceremony. A trophy was given to the team with the most points and was displayed in the employee break room. Awards were given to all nominees, and many award winners posted their certificates in their work spaces.

### Qualitative analysis of game evaluation survey

At the end of the game, a paper-based evaluation survey containing ten multiple choice and free-text response questions (supplemental Appendix E) was given to library employees during an all-staff meeting. Employees had a week to complete the game evaluation survey. Qualitative data were thematically coded according to Strauss and Corbin's method [46].

## RESULTS

Between 2016 and 2018, the University of Utah Resiliency Center conducted 3 surveys of Eccles Library employees. In 2016, only library faculty were eligible to participate in the survey, and the survey had an 80% response rate. In 2017, both library faculty and staff were eligible to participate, with response rates of 50% and 33%, respectively. In 2018, faculty and staff response rates were 93% and 54%, respectively.

Analysis of baseline demographics of faculty (in 2016) and staff (in 2017) showed no significant differences between employee types in sex, age, or race (Table 1). However, there was a significant difference between the proportions of faculty versus staff who reported being of Latino or Hispanic origin (*p*=0.0391). Specifically, 67% faculty members reported that they were not of Latino or Hispanic origin (with the others preferring not to disclose this information), whereas 100% of staff reported that they were not of Latino or Hispanic origin.

**Table 1:** Baseline demographics for faculty and staff

Demographics	Faculty (2016)		Staff (2017)		*p*-value
	n	(%)	n	(%)	
Total	12	(100.00%)	13	(100.00%)	
Sex					0.0535
Male	0	(—)	5	(38.46%)	
Female	10	(83.33%)	7	(53.85%)	
Prefer not to answer	1	(8.33%)	1	(7.69%)	
Race					0.1488
White	7	(58.33%)	11	(84.62%)	
Asian	1	(8.33%)	0	(—)	
Other	0	(—)	1	(7.69%)	
Prefer not to answer	4	(33.33%)	1	(7.69%)	
Hispanic or Latino origin					0.0391
No	8	(66.67%)	13	(100.00%)	
Prefer not to answer	4	(33.33%)	0	(—)	
Age (years)					
21–30	2	(16.67%)	1	(7.69%)	
31–40	3	(25.00%)	4	(30.77%)	
41–50	5	(41.67%)	2	(15.38%)	
51–64	2	(16.67%)	6	(46.15%)	

### Faculty survey results

Between 2016 and 2018, an increasing proportion of library faculty reported sufficient control over their workloads, corresponding with a decreasing proportion who reported burnout symptoms and job-related stress (Table 2). However, over time, fewer faculty reported overall job satisfaction, effectiveness of teamwork, and values aligned with leadership, and more reported a chaotic work atmosphere. These differences between years were not statistically significant, although the change in effective teamwork was associated with a large-sized effect.

**Table 2:** Comparison of American Medical Association (AMA) Mini-Z items for faculty between 2016 and 2018

Items	2016		2018		One-sided *p*-value	Cohen's h
	n	(%)	n	(%)		
Overall satisfied with job*					0.2792	0.291
Yes	9	(75.00%)	8	(61.54%)		
No	3	(25.00%)	5	(38.46%)		
Experiencing symptoms of burnout†					0.4740	0.065
Yes	5	(41.67%)	5	(38.46%)		
No	7	(58.33%)	8	(61.54%)		
Values align with leadership*					0.2690	0.291
Yes	9	(75.00%)	8	(61.54%)		
No	3	(25.00%)	5	(38.46%)		
Teams work efficiently together‡					0.1532	0.806
Yes	11	(100.00%)	11	(84.62%)		
No	0	(—)	2	(15.38%)		
Feeling great deal of job-related stress*					0.4449	0.090
Yes	7	(58.33%)	7	(53.85%)		
No	5	(41.67%)	6	(46.15%)		
Work atmosphere is hectic/chaotic§					0.2690	0.291
Yes	3	(25.00%)	5	(38.46%)		
No	9	(75.00%)	8	(61.54%)		
Sufficient control over workload‡					0.4409	0.229
Yes	8	(66.67%)	10	(76.92%)		
No	4	(33.33%)	3	(23.08%)		
Total	12	(100.00%)	13	(100.00%)		

* Yes: Strongly agree/agree; No: Neither agree nor disagree/disagree/strongly disagree.

† Yes: I am definitely burning out and have one or more symptoms of burnout, e.g., emotional exhaustion./The symptoms of burnout that I'm experiencing won't go away./I think about work frustrations a lot./I feel completely burned out. I am at the point where I may need to seek help. No: I enjoy my work. I have no symptoms of burnout./I am under stress and don't always have as much energy as I did, but I don't feel burned out.

‡ Yes: Optimal/good/satisfactory; No: Marginal/poor.

§ Yes: Hectic and chaotic/very busy; No: Busy, but reasonable/somewhat calm/calm.

### Staff survey results

Between 2017 and 2018, a decreasing proportion of library staff reported burnout, job-related stress, and a chaotic work atmosphere, and an increasing proportion reported sufficient control over their workload (Table 3). However, over time, fewer staff reported overall job satisfaction, values alignment with leadership, and effectiveness of teamwork. These differences between years were not statistically significant, although the changes in burnout and values alignment with leadership were associated with small-to-medium-sized effects.

**Table 3:** Comparison of AMA Mini-Z items for library staff between 2017 and 2018

Items	2017		2018		One-sided *p*-value	Cohen's h
	n	(%)	n	(%)		
Overall satisfied with job*					0.3727	0.213
Yes	11	(84.62%)	16	(76.19%)		
No	2	(15.38%)	5	(23.81%)		
Experiencing symptoms of burnout†					0.1749	0.376
Yes	8	(61.54%)	9	(42.86%)		
No	5	(38.46%)	12	(57.14%)		
Values align with leadership*					0.1488	0.425
Yes	11	(84.62%)	14	(66.67%)		
No	2	(15.38%)	7	(33.33%)		
Teams work efficiently together‡					0.7209	0.065
Yes	12	(92.31%)	19	(90.48%)		
No	1	(7.69%)	2	(9.52%)		
Feeling great deal of job-related stress*					0.5131	0.008
Yes	5	(38.46%)	8	(38.10%)		
No	8	(61.54%)	13	(61.90%)		
Work atmosphere is hectic/chaotic§					0.4125	0.107
Yes	5	(38.46%)	7	(33.33%)		
No	8	(61.54%)	14	(66.67%)		
Sufficient control over workload‡					0.6134	0.031
Yes	11	(84.62%)	18	(85.71%)		
No	2	(15.38%)	3	(14.29%)		
Total	13	(100.00%)	21	(100.00%)		

* Yes: Strongly agree/agree; No: Neither agree nor disagree/disagree/strongly disagree.

† Yes: I am definitely burning out and have one or more symptoms of burnout, e.g., emotional exhaustion./The symptoms of burnout that I'm experiencing won't go away./I think about work frustrations a lot./I feel completely burned out. I am at the point where I may need to seek help. No: I enjoy my work. I have no symptoms of burnout./I am under stress and don't always have as much energy as I did, but I don't feel burned out.

‡ Yes: Optimal/good/satisfactory; No: Marginal/poor.

§ Yes: Hectic and chaotic/very busy; No: Busy, but reasonable/somewhat calm/calm.

### Faculty versus staff survey results

In 2018, library staff scored better than library faculty on each of the AMA Mini-Z items, except the burnout question (Table 4). None of these differences were statistically significant, although differences in job satisfaction and job-related stress were associated with small-to-medium-sized effects. For the four open-ended questions concerning how the library should move forward to improve trust, success recognition, and support, employees provided suggestions related to transparent and open communication with less defensiveness, clearly defined strategic goals, and celebrations of success.

**Table 4:** Comparison of AMA Mini-Z items between library faculty and staff in 2018

Items	Faculty		Staff		One-sided *p*-value	Cohen's h
	n	(%)	n	(%)		
Overall satisfied with job*					0.2595	0.318
Yes	8	(61.54%)	16	(76.19%)		
No	5	(38.46%)	5	(23.81%)		
Experiencing symptoms of burnout†					0.4567	0.090
Yes	5	(38.46%)	9	(42.86%)		
No	8	(61.54%)	12	(57.14%)		
Values align with leadership*					0.7506	0.107
Yes	8	(61.54%)	14	(66.67%)		
No	5	(38.46%)	7	(33.33%)		
Teams work efficiently together‡					0.4125	0.179
Yes	11	(84.62%)	19	(90.48%)		
No	2	(15.38%)	2	(9.52%)		
Feeling great deal of job-related stress*					0.2958	0.317
Yes	7	(53.85%)	8	(38.10%)		
No	6	(46.15%)	13	(61.90%)		
Work atmosphere is hectic/chaotic§					0.4125	0.107
Yes	5	(38.46%)	7	(33.33%)		
No	8	(61.54%)	14	(66.67%)		
Sufficient control over workload‡					0.4177	0.227
Yes	10	(76.92%)	18	(85.71%)		
No	3	(23.08%)	3	(14.29%)		
Total	13	(100.00%)	21	(100.00%)		

* Yes: Strongly agree/agree; No: Neither agree nor disagree/disagree/strongly disagree.

† Yes: I am definitely burning out and have one or more symptoms of burnout, e.g., emotional exhaustion./The symptoms of burnout that I'm experiencing won't go away./I think about work frustrations a lot./I feel completely burned out. I am at the point where I may need to seek help. No: I enjoy my work. I have no symptoms of burnout./I am under stress and don't always have as much energy as I did, but I don't feel burned out.

‡ Yes: Optimal/good/satisfactory; No: Marginal/poor.

§ Yes: Hectic and chaotic/very busy; No: Busy, but reasonable/somewhat calm/calm.

### Qualitative analysis of game evaluation survey

Based on post-game survey data, the game design gave players control and improved team socializing. Three specific quotes from the post-game survey highlighted this flexibility and control: “I did this anyway, but it was nice to get ‘credit' for doing wellness activities”; “My own goals—no additional imposed goals”; and “Recording points for things I was doing anyways.” The social elements were specifically highlighted in the game design through diverse teams, physical score boards, and bonus points to address the concern of team collegiality. In the post-game survey, employees noted that they “met a few new people” and “Once in [the] staff lounge [I] was asked to join conversation.” Additionally, 70% of employees indicated that the game encouraged them to socialize with others.

Throughout the game, some team captains organized social events to garner bonus points. For example, a team “walked 11 stairs at 11 a.m.” We observed employees eating lunch together and walking around the library for quick discussions. Two team captains organized after-work hikes on Thursdays, and an estimated 6 employees participated. Twelve award nominations were submitted to the online survey. During the potluck lunch and awards ceremony, the winning team was presented with a large personalized trophy. The winning team had 7,312 points, beating the other teams by 5,047 points.

**Figure 1:**
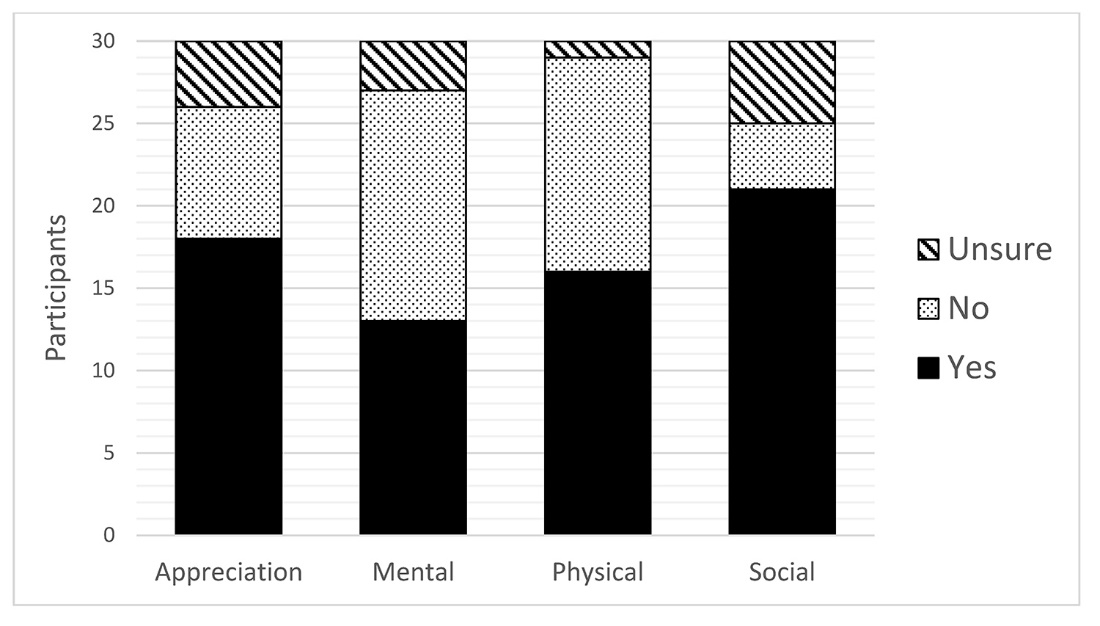
Number of employees reporting that the game encouraged them to do something for their wellness

Thirty out of the 59 employees (50.85%) completed the post-game survey. A total of 21 employees recorded their activity daily, 2–3 times per week, or weekly; whereas 2 employees recorded monthly and 5 employees recorded only once. More than half of employees found that the game encouraged them in the appreciation, physical, and social categories (Figure 1). After coding the qualitative data for “What did you enjoy most about the game?” free-text response question, 4 categories emerged: social, motivation, gameplay, and fun (Table 5). After coding the qualitative data for “What did you enjoy least about the game?” free-text response question, 3 categories emerged: game points, team engagement, and game duration (Table 6).

**Table 5:** Qualitative categories for “What did you enjoy most about the game?”

Category	n	Definition	Exemplar quotes
Social	19	Comments about the team-based game approach and the social and appreciation game components.	“I think this helped encourage people to socialize more, which was nice,” and “Team building.”
Motivation	6	Comments about how the game motivated them to learn about wellness or improve their wellness.	“Made me more aware of these health aspects” and “The motivation to make healthy choices.”
Gameplay	6	Comments concerning elements about gameplay that they enjoyed, such as no additional work, personalized goals, and habit recognition.	“Easy. No additional work,” and “Being able to think and track your wellness activities that you may or may not realize you do every day.”
Fun	5	Comments about fun aspect of the game.	“It… was fun. Thanks!”

**Table 6:** Qualitative categories for “What did you enjoy least about the game?”

Category	n	Definition	Exemplar quotes
Game points	10	Comments concerning the tracking system and a better defined point system.	“Tedious record keeping,” and “I think there could have been more defined way/types of points and maybe a cap per day,” and “Maybe online tracking with a dashboard of how teams are doing.”
Team engagement	7	Comments focused on low team engagement.	“My team did not do anything together,” “I did not hear from my team captain at all,” and “Encouraging team members to participate,” which focused on low team engagement.
Game duration	7	Comments stated that the game lasted too long.	“I thought it continued too long—[I] lost interest in recording,” and “Waiting three months for the ‘end.' Perhaps evaluating after one month and then switching groups would help keep motivation up.”

## DISCUSSION

The game intervention did not significantly impact burnout and job satisfaction but did create a more social environment and stimulated library employees to participate in wellness activities. To our knowledge, this was the first LIS study in which the same population of librarians was surveyed multiple times with validated burnout items and in which an intervention was created and assessed between surveys. Our results demonstrated that a game intervention might not be able to address the root causes of burnout but could encourage a more social environment.

The metrics for determining game success were the Resiliency Center's survey data, game participation, and a sense of community. According to the Resiliency Center's survey data, the game did not improve personal resilience or burnout. Participation was difficult to determine, as the post-game survey response was low (50.8%), and 7 employees indicated that they tracked their points only once a month or once at all. As 1 employee noted in the post-game survey, a “drop in participation” occurred near the end of the game. However, a sense of community was clearly developed during the course of the game. In the post-game survey, 70% indicated that the game encouraged them to do something for their social well-being. Additionally, 53% of the free-text comments for the question “What did you enjoy most about the game?” were coded to the social category. As mentioned earlier, the library employees ate lunch together, organized after-work hikes, and participated in more active meetings. Most employees (72%) indicated that they would play the game again. Therefore, the game met at least 1 of the 3 success metrics.

As LIS games primarily focus on education, health and corporate games were worth examining. One previous study at a corporate organization utilizing a team sport intervention to improve employee physical health and interpersonal communication found that employees who participated in the team sport intervention showed a 3% increase in interpersonal communication [33]. Similarly, our post-game survey respondents stated that they “met a few new people” and “Once in [the] staff lounge [I] was asked to join conversation.” Thus, both the previously described team sport intervention and our game increased socializing and communication among employees. As one post-game survey respondent stated, “There was more socializing. More ‘together' lunches. Some fun ‘walks' [and] ‘hikes' planned which build friendship.” Interestingly, another study on a weight loss mobile application (app) intervention found that the social support element encouraged more weight loss than the gamification element of the app [47]. These findings supported our decision in designing the game to focus on team collegiality and social aspects.

In addition to team collegiality and communication, we designed the game to allow flexibility due to concerns over faculty's perceived lack of control over their workloads in the 2016 burnout survey. Similar to Shahrestani and colleagues' health app intervention, we offered more flexibility for participants to record any activity [34]. Shahrestani and colleagues stated:

Unfortunately, daily steps only give a narrow view on activity levels…it may be detrimental in less controlled settings such as corporate environments where people are recruited to join health games and competitions. In such a setting, people who perform frequent swimming (or biking,…) activities would find it quite unfair if less fit challenge participants would systematically win the corporate challenges simply because they happen to make more steps per day. [34]

During the initial informal interviews, some library employees mentioned daily step count challenges as a possible intervention, which are very common in workplace settings. Shahrestani and colleagues' argument against just daily step counts [34] confirmed our reservations about daily step counts. Additionally, by avoiding required expectations and activities with the game, employees had flexibility and control over their involvement in activities. We anticipated that some employees would not engage or participate regardless of the intervention. By not being forced to participate or achieve specific requirements, such as step counts, employees maintained flexibility and control over their activities.

Another interesting finding in the present and previous studies were the differences in burnout depending on employee status. Eccles Library faculty experienced emotional exhaustion at a higher rate than library staff. Similarly, Shabani and colleagues found that employees with an LIS degree had higher levels of emotional exhaustion than library employees without an LIS degree [15]. At Eccles Library, an employee's status is usually determined by their education, so all library faculty have a master's degree in LIS, whereas staff rarely have an advanced degree in LIS. Thus, including a question concerning Eccles Library employees' highest level of education would have been helpful for making comparisons to Shabani and colleagues' findings. Additionally, Togia found that temporary employees experienced higher levels of emotional exhaustion than employees in permanent positions [13]. As a few full-time Eccles Library employees were on year-to-year contracts, it would be interesting to see if part-time and contract-based employees would also report higher levels of emotional exhaustion, similar to Togia's findings.

This study found more differences in employee status (faculty versus staff) and their work enjoyment, such as happiness at work, levels of stress, chaos, and values aligned with leaders. Despite working at the same library, the employee's status of faculty or staff influenced their satisfaction and fulfillment. The library faculty experienced a challenging work environment in 2017: five junior faculty left and two senior faculty members retired in less than one year. These numbers indicated that the leadership uncertainty and faculty turnover during 2017 might have impacted library faculty more than staff.

A variety of factors contribute to employee well-being, including both long-standing, predictable factors such as workload, layout of physical space, and job duties [1, 2, 21] and short-term factors such as leadership or employee turnover and group conflict [48–50]. Thus, addressing one factor in isolation may not be sufficient to change overall workplace well-being, thus limiting the impact of a targeted intervention. The lack of a major response to the game despite positive comments from participants, along with the reduction in burnout in February 2018 following a broader set of interventions, suggests a multifactorial approach was needed. Also, although games can be a useful strategy for encouraging a more social culture with fun activities, they have some potential limitations, including not addressing the root causes of burnout, taking too long, and having low rates of engagement. As noted by other studies, workplace demands and culture (e.g., personal, organizational, societal) may be barriers to participating in well-being activities at work [33, 51].

Employee burnout remains a concern at our library. However, although the game intervention did not improve burnout or job satisfaction, it did improve collegiality and recognition among employees. Thus, a game can encourage team-building but may not sufficiently address the root causes of burnout among health sciences librarians. LIS professionals and researchers should investigate Maslach's organizational level predictors of burnout [2, 21] to implement and assess other interventions aimed at addressing librarian burnout. A focus on vigor, dedication, and absorption may increase engagement and well-being [2]. A recent article uses Maslow's hierarchy of needs to describe an approach to interventions [52]. Individuals experiencing burnout should seek help from a health professional for guidance. There are increasing resources available to help institutions and individuals approach this situation, including the National Academy of Medicine Action Collaborative on Clinician Well-Being and Resilience [53] and the National Network of Libraries of Medicine's Wellness in the Library Workplace [54].

## SUPPLEMENTAL FILES

**Appendix A:** University of Utah health sciences faculty wellness needs assessment: survey of faculty wellness

**Appendix B:** University of Utah health sciences faculty wellness needs assessment: survey of faculty wellness, fall 2017

**Appendix C:** Spencer S. Eccles Health Sciences Library survey, winter 2018

**Appendix D:** Design box for the Spencer S. Eccles Health Sciences Library (EHSL) Wellness Game

**Appendix E:** Post-assessment of the Spencer S. Eccles Health Sciences Library Wellness Game
