# Supplementary material for: A workplace well-being game intervention for health sciences librarians to address burnout
Source: J Med Libr Assoc. 2020 Oct 1;108(4):605–17. doi: 10.5195/jmla.2020.742 (PMC7524620; doi:10.5195/jmla.2020.742)
Supplement: Supplementary file 1 — Appendix A: University of Utah health sciences faculty wellness needs assessment: survey of faculty wellness [file jmla-108-4-605-s01.pdf]

JMLA Journal of the Medical Library Association

SC
Supplemental content to
J Med Libr Assoc. Oct;108(4):dx.doi.org/10.5195/jmla.2020.742
www.jmla.mlanet.org
© Casucci, Locke, Henson, Qeadan 2020

A workplace well-being game intervention for health sciences librarians to address burnout

Tallie Casucci; Amy B. Locke; Autumn Henson; Fares Qeadan

APPENDIX A

University of Utah health sciences faculty wellness needs assessment: survey of faculty wellness

Faculty wellness, satisfaction, and prevention of burnout has become a top priority for the health system. In order to develop programs to optimize wellness, we are assessing faculty. This survey has two parts. The first looks at your level of burnout and possible related factors. The second looks at what we might do to improve faculty wellness.

We will use these data to present options to your departmental leadership and work with them to see what would be feasible to implement or focus on to create new programs or to strengthen existing programs.

Items with a "*" must be answered to complete the survey. Individual responses will not be tracked but will be collected in aggregate by department and division.

* 1. Primary department, program, or center

Other (please specify)

---

2. Division

Other (please specify)

---

3. Are you…?
- ○ Male
- ○ Female
- ○ Prefer not to answer

4. What is your age?
- ○ Under 21
- ○ 21–30
- ○ 31–40
- ○ 41–50
- ○ 51–64
- ○ 65+

SC
Supplemental content to
J Med Libr Assoc. Oct;108(4):dx.doi.org/10.5195/jmla.2020.742
www.jmla.mlanet.org
© Casucci, Locke, Henson, Qeadan 2020

5. Are you of Hispanic or Latino origin?
- O Yes
- O No
- O Prefer not to answer

6. What is your race?
- O Asian
- O Black or African American
- O American Indian or Alaska Native
- O Native Hawaiian or Other Pacific Islander
- O White
- O Prefer not to answer
- O Other (please specify)

---

*7. Overall, I am satisfied with my current job

Strongly disagree	Disagree	Neither agree nor disagree	Agree	Strongly agree
O	O	O	O	O

*8. I feel a great deal of stress because of my job

Strongly disagree	Disagree	Neither agree nor disagree	Agree	Strongly agree
O	O	O	O	O

*9. Using your own definition of "burnout," please select which of the following statements best describes you
- O I enjoy my work. I have no symptoms of burnout.
- O I am under stress and don't always have as much energy as I did, but I don't feel burned out.
- O I am definitely burning out and have one or more symptoms of burnout, e.g., emotional exhaustion.
- O The symptoms of burnout that I'm experiencing won't go away. I think about work frustrations a lot.
- O I feel completely burned out. I am at the point where I may need to seek help.

*10. My control over my workload is

Poor	Marginal	Satisfactory	Good	Optimal
O	O	O	O	O

*11. On the scale below, please indicate which number best describes the atmosphere in your primary work area

Calm	Somewhat calm	Busy, but reasonable	Very busy	Hectic and chaotic
O	O	O	O	O

*12. My professional values are well aligned with those of my organizational leaders

Strongly disagree	Disagree	Neither agree nor disagree	Agree	Strongly agree
O	O	O	O	O

*13. Do you see patients?
- O Yes
- O No

SC
Supplemental content to
J Med Libr Assoc. Oct;108(4):dx.doi.org/10.5195/jmla.2020.742
www.jmla.mlanet.org
© Casucci, Locke, Henson, Qeadan 2020

*14. The degree to which my care team works efficiently together is

Poor	Marginal	Satisfactory	Good	Optimal
O	O	O	O	O

*15. Sufficiency of time for documentation is

Poor	Marginal	Satisfactory	Good	Optimal
O	O	O	O	O

16. How many hours do you spend on work per week, including hours spent outside the office or hospital?
- O Less than 20
- O 20–29
- O 30–39
- O 40–49
- O 50–59
- O 60–69
- O 70–80
- O More than 80

17. What is your percent full-time equivalent (FTE)?
- O Less than 0.50
- O 0.50–0.74
- O 0.75–0.95
- O 1.0

18. In what type of setting do you spend the majority of your clinical time?
- O Inpatient
- O Outpatient
- O Both inpatient and outpatient
- O Other (please specify)

---

19. How many hours of direct OUTPATIENT care do you provide during a typical week of practice?
- O 100–80 hours
- O 60–80 hours
- O 40–20 hours
- O 20–10 hours
- O Less than 10 hours
- O I do not provide direct outpatient care
- O Don't know

20. If you personally provide outpatient care, what is the average number of patients you see in a four-hour session?
- O Less than 4
- O 4–5
- O 6–8
- O 9–10
- O 11–12
- O 13–15
- O 16–20

JMLA Journal of the Medical Library Association

SC
Supplemental content to
J Med Libr Assoc. Oct;108(4):dx.doi.org/10.5195/jmla.2020.742
www.jmla.mlanet.org
© Casucci, Locke, Henson, Qeadan 2020

- O More than 20
- O N/A

21. How many hours of direct INPATIENT care do you provide during a typical week of practice?
- O 100–80 hours
- O 60–80 hours
- O 40–20 hours
- O Less than 20 hours
- O I do not provide direct inpatient care
- O Don't know

22. If you provide inpatient care, what is your average daily census?
- O Less than 5
- O 5–10
- O 11–15
- O 16–20
- O 21–25
- O 26–30
- O 31–35
- O 36–40
- O 41–45
- O More than 45
- O N/A

*23. The amount of time I spend on the electronic medical record (EMR) at home is

Minimal/none	Modest	Satisfactory	Moderately high	Excessive
O	O	O	O	O

*24. My proficiency with EMR use is

Poor	Marginal	Satisfactory	Good	Optimal
O	O	O	O	O

*25. I have recently experienced feelings of anxiety, depression, or irritability about practicing medicine

Strongly disagree	Disagree	Neither agree nor disagree	Agree	Strongly agree
O	O	O	O	O

*26. Please indicate the extent to which you feel supported/appreciated by each of the following groups:

	Strongly disagree	Disagree	Neither agree nor disagree	Agree	Strongly agree
Peers	O	O	O	O	O
Patients	O	O	O	O	O
Patients' families	O	O	O	O	O
Immediate supervisor/ department chair	O	O	O	O	O

JMLA Journal of the Medical Library Association

SC
Supplemental content to
J Med Libr Assoc. Oct;108(4):dx.doi.org/10.5195/jmla.2020.742
www.jmla.mlanet.org
© Casucci, Locke, Henson, Qeadan 2020

27. Which three of the following are the main sources of any dissatisfaction you may have experienced with practicing medicine? Please select up to three options

- ☐ Electronic health record (EHR)/EMR
- ☐ Workplace chaos
- ☐ Insufficient staffing
- ☐ Billing and coding
- ☐ Meaningful use requirements prior authorizations
- ☐ Lack of control of work and work environment
- ☐ Excessive workload
- ☐ Lack of meaningful work
- ☐ Other (please specify)

---

28. Which three of the following would improve your satisfaction with practicing medicine? Please select up to three options

- ☐ Improved practice efficiency and better defined workflows
- ☐ Reduced time spent on non-patient-care activities
- ☐ Ability to delegate clerical tasks to trusted support staff
- ☐ Ability to spend more time with patients and/or their families during appointments
- ☐ More time for continuing education and/or research
- ☐ Improved decision support
- ☐ Ability to network more with peers
- ☐ Other (please specify)

---

29. You indicated the following are sources of dissatisfaction you may have experienced with practicing medicine. Please rank the following with #1 being your primary source of dissatisfaction

- ☐ EHR/EMR
- ☐ Workplace chaos
- ☐ Insufficient staffing
- ☐ Billing and coding
- ☐ Meaningful use requirements prior authorizations
- ☐ Lack of control of work and work environment
- ☐ Excessive workload
- ☐ Lack of meaningful work

30. Which three of the following would improve your satisfaction with practicing medicine? Please rank the following with #1 being the item that would most improve your satisfaction

- ☐ Improved practice efficiency and better defined workflows
- ☐ Reduced time spent on non-patient-care activities
- ☐ Ability to delegate clerical tasks to trusted support staff
- ☐ Ability to spend more time with patients and/or their families during appointments
- ☐ More time for continuing education and/or research
- ☐ Improved decision support
- ☐ Ability to network more with peers
- ☐ Other (please specify)

SC
Supplemental content to
J Med Libr Assoc. Oct;108(4):dx.doi.org/10.5195/jmla.2020.742
www.jmla.mlanet.org
© Casucci, Locke, Henson, Qeadan 2020

31. Rate your level of clinical support (includes master's [MA] and nurse support)

Poor	Marginal	Satisfactory	Good	Optimal
O	O	O	O	O

32. How many years have you been in practice since completing residency?
- O 1–5 years
- O 6–10 years
- O 11–15 years
- O 16–20 years
- O More than 20 years

33. In which of the following ways do you document clinical information in your practice? Please select all that apply
- O Team documentation/scribe
- O Dictation to transcription
- O Dictation to voice recognition
- O I type my notes and/or use templates
- O Other (please specify)

---

*34. What is your terminal degree: medical degree/doctor of osteopathy (MD/DO), physician assistant (PA), advanced practice registered nurse (APRN), doctorate (PhD), doctor of pharmacy (PharmD), other (can choose more than one)
- O MD/DO
- O PA
- O APRN
- O PhD
- O Pharm D
- O Other (please specify)

---

*35. The degree to which my team works efficiently together is

Poor	Marginal	Satisfactory	Good	Optimal
O	O	O	O	O

*36. I have recently experienced feelings of anxiety, depression, or irritability about my work

Strongly disagree	Disagree	Neither agree nor disagree	Agree	Strongly agree
O	O	O	O	O

37. Please indicate the extent to which you feel supported/appreciated by each of the following groups

Peers	O	O	O	O	O
Immediate supervisor/department chair	O	O	O	O	O

38. Which three of the following are the main sources of any dissatisfaction you may have experienced in your role in academics? Please select up to three options
- O Workplace chaos
- O Insufficient staffing

Supplemental content to
J Med Libr Assoc. Oct;108(4):dx.doi.org/10.5195/jmla.2020.742
www.jmla.mlanet.org
© Casucci, Locke, Henson, Qeadan 2020

- ○ Lack of control of work and work environment
- ○ Excessive workload
- ○ Lack of meaningful work
- ○ Other (please specify)

---

39. Which three of the following would improve your satisfaction with your academic roles? Please select up to three options

- ○ Improved team efficiency and better defined workflows
- ○ Ability to delegate clerical tasks to trusted support staff
- ○ More time for continuing education and/or research
- ○ Ability to network more with peers
- ○ Other (please specify)

---

40. You indicated the following are sources of dissatisfaction you may have experienced in your role in academics. Please rank the following with #1 being your primary source of dissatisfaction

- ☐ Workplace chaos
- ☐ Insufficient staffing
- ☐ Lack of control of work and work environment
- ☐ Excessive workload
- ☐ Lack of meaningful work

41. You indicated the following would improve your satisfaction with academic roles. Please rank the following with #1 being the item that would most improve your satisfaction

- ☐ Improved team efficiency and better defined workflows
- ☐ Ability to delegate clerical tasks to trusted support staff
- ☐ More time for continuing education and/or research
- ☐ Ability to network more with peers

42. How many hours do you spend on work per week, including hours spent outside the office?

- ○ Less than 20
- ○ 20–29
- ○ 30–39
- ○ 40–49
- ○ 50–59
- ○ 60–69
- ○ 70–80
- ○ More than 80

43. What is your percent FTE?

- ○ Less than 0.50
- ○ 0.50–0.74
- ○ 0.75–0.95
- ○ 1.0

44. How many years since you completed your training?

- ○ 1–5 years
- ○ 6–10 years

- O 11–15 years
- O 16–20 years
- O More than 20 years

*45. What is your terminal degree: MD/DO, PA, APRN, PHD, Pharm D, other (can choose more than one)
- O MD/DO
- O PA
- O APRN
- O PhD
- O Pharm D
- O Other (please specify)

Now that we've asked about your level of burnout and possible causes, we would like your opinion on what would be most useful to you as a part of a program to optimize wellness and faculty satisfaction.

46. Leadership training/faculty development sessions focused on

	Minimally valuable	Somewhat valuable	Neutral	Valuable	Extremely valuable
Optimizing career trajectory	O	O	O	O	O
Work-life balance	O	O	O	O	O
Time management/ prioritization/ delegation	O	O	O	O	O
Mindfulness training	O	O	O	O	O
Session(s) on finding meaning and purpose	O	O	O	O	O

47. If you find value in one or more of these sessions, when would be the most appropriate time to offer them? Please select all that apply
- O During regularly scheduled meetings
- O Department specific retreats
- O Evenings and weekends
- O Specific programs offered to interested faculty, such as through the Faculty Development Office

48. Structured mentorship program

Minimally valuable	Somewhat valuable	Neutral	Valuable	Extremely valuable
O	O	O	O	O

49. Flexible work hours

Minimally valuable	Somewhat valuable	Neutral	Valuable	Extremely valuable
O	O	O	O	O

50. Support for hiring/retaining part time faculty

Minimally valuable	Somewhat valuable	Neutral	Valuable	Extremely valuable
O	O	O	O	O

Supplemental content to
J Med Libr Assoc. Oct;108(4):dx.doi.org/10.5195/jmla.2020.742
www.jmla.mlanet.org
© Casucci, Locke, Henson, Qeadan 2020

51. Develop a structure to recognize and quantify academic work/educational efforts and reimburse or reward accordingly (similar to the time banking program at Stanford)

Minimally valuable	Somewhat valuable	Neutral	Valuable	Extremely valuable
○	○	○	○	○

52. Informational sessions about faculty mental health and available resources

Minimally valuable	Somewhat valuable	Neutral	Valuable	Extremely valuable
○	○	○	○	○

53. Onsite child care

Minimally valuable	Somewhat valuable	Neutral	Valuable	Extremely valuable
○	○	○	○	○

54. Focus on healthy foods at meetings/conferences

Minimally valuable	Somewhat valuable	Neutral	Valuable	Extremely valuable
○	○	○	○	○

55. Educational sessions on nutrition/healthy eating

Minimally valuable	Somewhat valuable	Neutral	Valuable	Extremely valuable
○	○	○	○	○

56. Onsite exercise facilities

Minimally valuable	Somewhat valuable	Neutral	Valuable	Extremely valuable
○	○	○	○	○

57. Increased bike-ability and walkability of workplace

Minimally valuable	Somewhat valuable	Neutral	Valuable	Extremely valuable
○	○	○	○	○

58. Active workstations (e.g., treadmill desk)

Minimally valuable	Somewhat valuable	Neutral	Valuable	Extremely valuable
○	○	○	○	○

59. Department sponsored physically active group sessions for team building and personal wellness

Minimally valuable	Somewhat valuable	Neutral	Valuable	Extremely valuable
○	○	○	○	○

60. Efforts to improve the physical space of your work environment

Minimally valuable	Somewhat valuable	Neutral	Valuable	Extremely valuable
○	○	○	○	○

SC
Supplemental content to
J Med Libr Assoc. Oct;108(4):dx.doi.org/10.5195/jmla.2020.742
www.jmla.mlanet.org
© Casucci, Locke, Henson, Qeadan 2020

61. Please rate the importance of improving the following

	Minimally valuable	Somewhat valuable	Neutral	Valuable	Extremely valuable
Natural light	O	O	O	O	O
Quiet space to work	O	O	O	O	O
Updated equipment	O	O	O	O	O
Décor	O	O	O	O	O
Other (please specify)					

---

62. Guided quality improvement projects or pilots to focus on decreasing provider work (example: Epic work flow, scheduling, optimizing staff numbers and tasks)

Minimally valuable	Somewhat valuable	Neutral	Valuable	Extremely valuable
O	O	O	O	O

63. Faculty development sessions focused on efficiency with Epic

Minimally valuable	Somewhat valuable	Neutral	Valuable	Extremely valuable
O	O	O	O	O

64. Assistance with grant writing/submission

Minimally valuable	Somewhat valuable	Neutral	Valuable	Extremely valuable
O	O	O	O	O

65. Assistance with academic writing

Minimally valuable	Somewhat valuable	Neutral	Valuable	Extremely valuable
O	O	O	O	O

66. Assistance with seeking philanthropy for programs

Minimally valuable	Somewhat valuable	Neutral	Valuable	Extremely valuable
O	O	O	O	O

67. What are the top 1–3 issues you feel are essential for us to address for optimal wellness and satisfaction?

---

68. Are there other ideas you have to improve faculty wellness and satisfaction?
