# Supplementary material for: A workplace well-being game intervention for health sciences librarians to address burnout
Source: J Med Libr Assoc. 2020 Oct 1;108(4):605–17. doi: 10.5195/jmla.2020.742 (PMC7524620; doi:10.5195/jmla.2020.742)
Supplement: Supplementary file 2 — Appendix B: University of Utah health sciences faculty wellness needs assessment: survey of faculty wellness, fall 2017 [file jmla-108-4-605-s02.pdf]

Journal of the Medical Library Association

Supplemental content to
J Med Libr Assoc. Oct;108(4):dx.doi.org/10.5195/jmla.2020.742
www.jmla.mlanet.org
© Casucci, Locke, Henson, Qeadan 2020

A workplace well-being game intervention for health sciences librarians to address burnout

Tallie Casucci; Amy B. Locke; Autumn Henson; Fares Qeadan

APPENDIX B

University of Utah health sciences faculty wellness needs assessment: survey of faculty wellness, fall 2017

Faculty and staff wellness, satisfaction, and prevention of burnout are top priorities for the health system. In order to continue to develop programs to optimize wellness, the Resiliency Center and the Office of Wellness and Integrative Health are assessing faculty and staff. This survey is estimated to take ten minutes.

Items with a "*" must be answered to complete the survey. This survey is anonymous; individual responses will not be tracked but will be collected in aggregate by department and division. Any question with less than five responses will not be reported to leadership.

*1. Are you...?

- ○ Faculty
- ○ Staff

*2. What is your clinical full-time equivalent (FTE)?

- ○ < 0.2
- ○ 0.2–0.4
- ○ 0.41–0.6
- ○ 0.61–0.8
- ○ 0.8
- ○ N/A

*3. Are you a/an...? (multiple answers acceptable)

- ○ Advanced practice registered nurse (APRN)
- ○ Dietitian
- ○ Master's
- ○ Medical doctor/doctor of osteopathy (MD/DO)
- ○ Occupational therapist (OT)
- ○ Physician assistant (PA)
- ○ Doctor of pharmacy (PharmD)
- ○ Doctorate (PhD)
- ○ Physical therapist (PT)
- ○ Registered nurse (RN)
- ○ Clinical social worker (CSW)/licensed clinical social worker (LCSW)
- ○ Other (please specify)

JMLA Journal of the Medical Library Association

SC
Supplemental content to
J Med Libr Assoc. Oct;108(4):dx.doi.org/10.5195/jmla.2020.742
www.jmla.mlanet.org
© Casucci, Locke, Henson, Qeadan 2020

*4. Are you...?
- O Tenure line
- O Career line: clinical
- O Career line: lecturer
- O Career line: research
- O Adjunct
- O Visiting

5. What is your academic rank?
- O Assistant professor
- O Associate professor
- O Instructor
- O Professor
- O Other (please specify)

---

6. Highest degree attained?
- O High school
- O Associate's degree
- O Bachelor's degree
- O Master's degree
- O Doctorate degree
- O Other (please specify)

---

7. What is your primary role?
- O Administration
- O APRN
- O Attorney
- O Call center/dispatch
- O Clerical
- O Clinical psychology
- O Dental
- O Dietetics
- O Facilities/environmental services
- O Family therapy
- O Finance
- O Food service
- O General management
- O Human resources
- O Information technology
- O MD/DO
- O Medical assistant
- O Nurse
- O Nursing assistant
- O Paramedic
- O Patient transport
- O Physical therapy
- O Physician assistant

Journal of the Medical Library Association

Supplemental content to
J Med Libr Assoc. Oct;108(4):dx.doi.org/10.5195/jmla.2020.742
www.jmla.mlanet.org
© Casucci, Locke, Henson, Qeadan 2020

- Occupational therapy
- Technician (includes pharmacy, laboratory, radiology, and other patient care roles)
- Social work
- Speech/language therapy
- Other (please specify)

---

*8. Primary college, school, or location

If Other, please specify

---

*9. College of Health primary department or program

If Other, please specify

---

*10. College of Nursing primary department, program, or center

---

If Other, please specify

---

*11. College of Pharmacy primary department, program, or center

---

If Other, please specify

---

*12. Huntsman Cancer Institute primary department, program, or center

---

If Other, please specify

---

*13. School of medicine primary department, program, or center

---

If Other, please specify

---

14. School of Medicine division

If Other, please specify

---

*15. Other health sciences primary department, program, or center

---

If Other, please specify

Supplemental content to
J Med Libr Assoc. Oct;108(4):dx.doi.org/10.5195/jmla.2020.742
www.jmla.mlanet.org
© Casucci, Locke, Henson, Qeadan 2020

16. Overall, I am satisfied with my current job

Strongly disagree	Disagree	Neither agree nor disagree	Agree	Strongly agree
○	○	○	○	○

*17. Using your own definition of "burnout," please select which of the following statements best describes you

○ I enjoy my work. I have no symptoms of burnout.
○ I am under stress and don't always have as much energy as I did, but I don't feel burned out.
○ I am definitely burning out and have one or more symptoms of burnout, e.g., emotional exhaustion.
○ The symptoms of burnout that I'm experiencing won't go away. I think about work frustrations a lot.
○ I feel completely burned out. I am at the point where I may need to seek help.

*18. My professional values are well aligned with those of my organizational leaders

Strongly disagree	Disagree	Neither agree nor disagree	Agree	Strongly agree
○	○	○	○	○

*19. The degree to which my team works efficiently together is

Poor	Marginal	Satisfactory	Good	Optimal
○	○	○	○	○

*20. I feel a great deal of stress because of my job

Strongly disagree	Disagree	Neither agree nor disagree	Agree	Strongly agree
○	○	○	○	○

*21. On the scale below, please indicate which number best describes the atmosphere in your primary work area

Calm	Somewhat calm	Busy, but reasonable	Very busy	Hectic and chaotic
○	○	○	○	○

*22. My control over my workload is

Poor	Marginal	Satisfactory	Good	Optimal
○	○	○	○	○

*23. How often do you feel you've become more callous toward people since you took this job?

○ Never
○ A few times a year or less
○ Once a month or less
○ A few times a month
○ Once a week
○ A few times a week
○ Every day

*24. I feel happy at work

Not at all true	Somewhat true	Moderately true	Very true	Completely true
○	○	○	○	○

*25. I feel worthwhile at work

Not at all true	Somewhat true	Moderately true	Very true	Completely true
○	○	○	○	○

SC
Supplemental content to
J Med Libr Assoc. Oct;108(4):dx.doi.org/10.5195/jmla.2020.742
www.jmla.mlanet.org
© Casucci, Locke, Henson, Qeadan 2020

*26. I find meaning in my work

Not at all true	Somewhat true	Moderately true	Very true	Completely true
O	O	O	O	O

*27. I would recommend the University of Utah to my friends as a great place to work

Strongly disagree	Disagree	Neither agree nor disagree	Agree	Strongly agree
O	O	O	O	O

*28. Do you plan to leave the University of Utah in the next 1–2 years?
- O Yes, I am seriously considering leaving this university in the next 1–2 years
- O No, I plan on staying at least that long
- O Don't know

29. Over the past 2 weeks, how often have you had little interest or pleasure in doing things?
- O Not at all
- O Several days
- O More than half of the days
- O Nearly every day

30. Over the past 2 weeks, how often have you been feeling down, depressed, or hopeless?
- O Not at all
- O Several days
- O More than half of the days
- O Nearly every day

31. In the past 12 months, about how many days did you miss work because of illness or injury (do not include parental leave)?
- O 0 days
- O 1–2 days
- O 3–5 days
- O 6–10 days
- O More than 10 days

*32. Do you provide clinical care?
- O Yes
- O No

*33. The amount of time I spend on the electronic medical record (EMR) at home is

Minimal/none	Modest	Satisfactory	Moderately high	Excessive
O	O	O	O	O

*34. Sufficiency of time for documentation is

Poor	Marginal	Satisfactory	Good	Optimal
O	O	O	O	O

35. Have you experienced a patient safety event within the past year that caused personal problems, such as anxiety, depression, or concerns about your ability to perform your job?
- O Yes
- O No

JMLA Journal of the Medical Library Association

Supplemental content to
J Med Libr Assoc. Oct;108(4):dx.doi.org/10.5195/jmla.2020.742
www.jmla.mlanet.org
© Casucci, Locke, Henson, Qeadan 2020

36. If so, did you receive institutional support to assist you with this stress?
- O Yes
- O No
- O N/A

37. How many hours do you spend on work per week, including hours spent outside the office or hospital?
- O Less than 20
- O 20–29
- O 30–39
- O 40–49
- O 50–59
- O 60–69
- O 70–80
- O More than 80

38. How many years since you completed your training?
- O 1–5 years
- O 6–10 years
- O 11–15 years
- O 16–20 years
- O More than 20 years

39. Are you…?
- O Male
- O Female
- O Prefer not to answer

40. What is your age?
- O Under 21
- O 21–30
- O 31–40
- O 41–50
- O 51–64
- O 65+

41. What is your race?
- O Asian
- O Black or African American
- O American Indian or Alaska Native
- O Native Hawaiian or Other Pacific Islander
- O White
- O Prefer not to answer
- O Other (please specify)

JMLA Journal of the Medical Library Association

SC
Supplemental content to
J Med Libr Assoc. Oct;108(4):dx.doi.org/10.5195/jmla.2020.742
www.jmla.mlanet.org
© Casucci, Locke, Henson, Qeadan 2020

42. Are you of Hispanic or Latino origin?
- O Yes
- O No
- O Prefer not to answer

Now that we've asked about your level of burnout and possible causes, we would like your opinion on what would be most useful to you as a part of a program to optimize wellness and satisfaction.

43. Communication skills training, such as working with difficult patients, clients, colleagues, peers, etc.

Minimally valuable	Somewhat valuable	Neutral	Valuable	Extremely valuable
O	O	O	O	O

44. Mindfulness classes

Minimally valuable	Somewhat valuable	Neutral	Valuable	Extremely valuable
O	O	O	O	O

45. Self-care retreats

Minimally valuable	Somewhat valuable	Neutral	Valuable	Extremely valuable
O	O	O	O	O

46. Peer support program

Minimally valuable	Somewhat valuable	Neutral	Valuable	Extremely valuable
O	O	O	O	O

47. Quality improvement projects focused on wellness

Minimally valuable	Somewhat valuable	Neutral	Valuable	Extremely valuable
O	O	O	O	O

48. Clinic flow and efficiency projects

Minimally valuable	Somewhat valuable	Neutral	Valuable	Extremely valuable
O	O	O	O	O

49. Support for team led projects targeted specifically to the needs of your group

Minimally valuable	Somewhat valuable	Neutral	Valuable	Extremely valuable
O	O	O	O	O

50. Institutional support for part time work or more flexible schedule

Minimally valuable	Somewhat valuable	Neutral	Valuable	Extremely valuable
O	O	O	O	O

51. What ideas do you have to improve faculty and staff wellness and satisfaction?

If you feel you or someone else needs help with burnout or other concerns, please contact Employee Assistance Program (EAP) at 801.587.9319 or the Resiliency Center staff at resiliencycenter@hsc.utah.edu.

Thank you for helping us make the University of Utah a place where faculty and staff are passionate about and energized by work.

uofuhealth.org/resiliencycenter
