# Supplementary material for: A workplace well-being game intervention for health sciences librarians to address burnout
Source: J Med Libr Assoc. 2020 Oct 1;108(4):605–17. doi: 10.5195/jmla.2020.742 (PMC7524620; doi:10.5195/jmla.2020.742)
Supplement: Supplementary file 3 — Appendix C: Spencer S. Eccles Health Sciences Library survey, winter 2018 [file jmla-108-4-605-s03.pdf]

JMLA Journal of the Medical Library Association

SC
Supplemental content to
J Med Libr Assoc. Oct;108(4):dx.doi.org/10.5195/jmla.2020.742
www.jmla.mlanet.org
© Casucci, Locke, Henson, Qeadan 2020

A workplace well-being game intervention for health sciences librarians to address burnout

Tallie Casucci; Amy B. Locke; Autumn Henson; Fares Qeadan

APPENDIX C

Spencer S. Eccles Health Sciences Library survey, winter 2018

Faculty and staff wellness, satisfaction, and prevention of burnout are top priorities for the health system. In order to continue to develop programs to optimize wellness, the Resiliency Center and the Office of Wellness and Integrative Health are partnering with EHSL faculty and staff.

This survey is estimated to take ten minutes.

Items with a "*" must be answered in order to complete the survey. This survey is anonymous; individual responses will not be tracked but will be collected in aggregate by department and division. Any question with less than five responses will not be reported to leadership.

*1. Are you?
- O Faculty
- O Staff

*2. Overall, I am satisfied with my current job

Strongly disagree	Disagree	Neither agree nor disagree	Agree	Strongly agree
O	O	O	O	O

*3. Using your own definition of "burnout," please select which of the following statements best describes you
- O I enjoy my work. I have no symptoms of burnout.
- O I am under stress and don't always have as much energy as I did, but I don't feel burned out.
- O I am definitely burning out and have one or more symptoms of burnout, e.g., emotional exhaustion.
- O The symptoms of burnout that I'm experiencing won't go away. I think about work frustrations a lot.
- O I feel completely burned out. I am at the point where I may need to seek help.

*4. My professional values are well aligned with those of my organizational leaders

Strongly disagree	Disagree	Neither agree nor disagree	Agree	Strongly agree
O	O	O	O	O

*5. The degree to which my team works efficiently together is

Poor	Marginal	Satisfactory	Good	Optimal
O	O	O	O	O

*6. I feel a great deal of stress because of my job

Strongly disagree	Disagree	Neither agree nor disagree	Agree	Strongly agree
O	O	O	O	O

JMLA Journal of the Medical Library Association

SC
Supplemental content to
J Med Libr Assoc. Oct;108(4):dx.doi.org/10.5195/jmla.2020.742
www.jmla.mlanet.org
© Casucci, Locke, Henson, Qeadan 2020

*7. Please indicate what description best describes the atmosphere in your primary work area

Calm	Somewhat calm	Busy, but reasonable	Very busy	Hectic	Chaotic
○	○	○	○	○	○

*8. My control over my workload is

Poor	Marginal	Satisfactory	Good	Optimal
○	○	○	○	○

*9. How often do you feel you've become more callous toward people since you took this job?
- ○ Never
- ○ A few times a year or less
- ○ Once a month or less
- ○ A few times a month
- ○ Once a week
- ○ A few times a week
- ○ Every day

*10. I feel happy at work

Not at all true	Somewhat true	Moderately true	Very true	Completely true
○	○	○	○	○

*11. I feel worthwhile at work

Not at all true	Somewhat true	Moderately true	Very true	Completely true
○	○	○	○	○

*12. I find meaning in my work

Not at all true	Somewhat true	Moderately true	Very true	Completely true
○	○	○	○	○

*13. I would recommend the University of Utah to my friends as a great place to work

Not at all true	Somewhat true	Moderately true	Very true	Completely true
○	○	○	○	○

*14. Do you plan to leave the university in the next 1–2 years?
- ○ Yes, I am seriously considering leaving this university in the next 1–2 years.
- ○ No, I plan on staying at least that long
- ○ I don't know

15. Over the past 2 weeks, how often have you been bothered by any of the following problems?

	Not at all	Several days	More than half of the days	Nearly every day
Little interest or pleasure in doing things	○	○	○	○
Feeling down, depressed, or hopeless	○	○	○	○

16. In the past 12 months, about how many days did you miss work because of illness or injury (do not include maternity leave)?

SC
Supplemental content to
J Med Libr Assoc. Oct;108(4):dx.doi.org/10.5195/jmla.2020.742
www.jmla.mlanet.org
© Casucci, Locke, Henson, Qeadan 2020

17. How many hours do you spend on work per week, including hours spent outside the office or hospital?
- O Less than 20
- O 20–29
- O 30–39
- O 40–49
- O 50–59
- O 60–69
- O 70–79
- O More than 80

Now that we've asked about your level of burnout and possible causes, we would like your opinion on what would be most useful to you as a part of a program to optimize wellness and satisfaction.

18. Please rate the following:

	Minimally valuable	Somewhat valuable	Neutral	Valuable	Extremely valuable
Mentorship/career planning assistance	O	O	O	O	O
Sessions on work-life integration or balance	O	O	O	O	O
Communication skills training, such as working with difficult patients	O	O	O	O	O
Mindfulness classes	O	O	O	O	O
Self-care retreats	O	O	O	O	O
Peer support program	O	O	O	O	O
Quality improvement projects focused on wellness	O	O	O	O	O
Support for team-led projects targeted specifically to the needs of your group	O	O	O	O	O
Institutional support for part-time work or more flexible schedule	O	O	O	O	O
Team building activities to improve collegiality of group	O	O	O	O	O

19. What can everyone at the library do to rebuild a culture of trust?

---

20. What would an environment built on trust look like?

---

21. How can leadership and peers better recognize our successes and accomplishments?

JMLA Journal of the Medical Library Association

Supplemental content to
J Med Libr Assoc. Oct;108(4):dx.doi.org/10.5195/jmla.2020.742
www.jmla.mlanet.org
© Casucci, Locke, Henson, Qeadan 2020

22. How can the Resiliency Center or the leadership of EHSL better support faculty and staff wellness/satisfaction in any ways not already mentioned?

If you feel you or someone else needs help with burnout or other concerns, please contact Employee Assistance Program (EAP) at 801.587.9319 or the Resiliency Center staff at resiliencycenter@hsc.utah.edu.
