# Supplementary material for: A workplace well-being game intervention for health sciences librarians to address burnout
Source: J Med Libr Assoc. 2020 Oct 1;108(4):605–17. doi: 10.5195/jmla.2020.742 (PMC7524620; doi:10.5195/jmla.2020.742)

JMLA Journal of the Medical Library Association

SC
Supplemental content to
J Med Libr Assoc. Oct;108(4):dx.doi.org/10.5195/jmla.2020.742
www.jmla.mlanet.org
© Casucci, Locke, Henson, Qeadan 2020

A workplace well-being game intervention for health sciences librarians to address burnout

Tallie Casucci; Amy B. Locke; Autumn Henson; Fares Qeadan

APPENDIX D

Design box for the Spencer S. Eccles Health Sciences Library (EHSL) Wellness Game

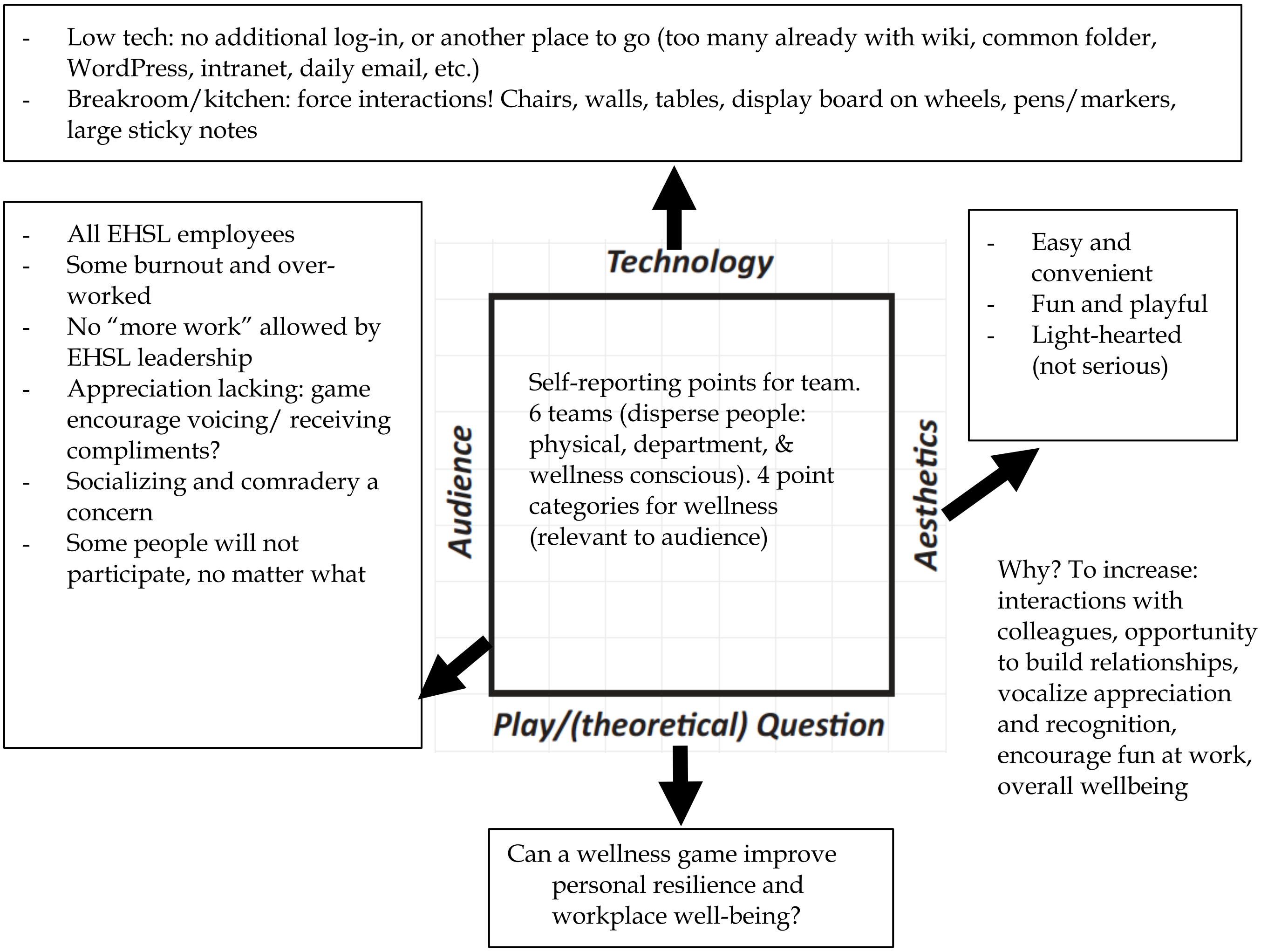

Supplement: Supplementary file 4 — Appendix D: Design box for the Spencer S. Eccles Health Sciences Library (EHSL) Wellness Game [file jmla-108-4-605-s04.pdf]
