# Supplementary material for: A workplace well-being game intervention for health sciences librarians to address burnout
Source: J Med Libr Assoc. 2020 Oct 1;108(4):605–17. doi: 10.5195/jmla.2020.742 (PMC7524620; doi:10.5195/jmla.2020.742)
Supplement: Supplementary file 5 — Appendix E: Post-assessment of the Spencer S. Eccles Health Sciences Library Wellness Game [file jmla-108-4-605-s05.pdf]

JMLA Journal of the Medical Library Association

Supplemental content to
J Med Libr Assoc. Oct;108(4):dx.doi.org/10.5195/jmla.2020.742
www.jmla.mlanet.org
© Casucci, Locke, Henson, Qeadan 2020

A workplace well-being game intervention for health sciences librarians to address burnout

Tallie Casucci; Amy B. Locke; Autumn Henson; Fares Qeadan

APPENDIX E

Post-assessment of the Spencer S. Eccles Health Sciences Library Wellness Game

Please answer the following questions by circling the choice option or supplying your own written response. This information will remain anonymous. There are no incentives to complete this survey.

1. How frequently did you record your participation in the EHSL Wellness Game?
   Daily / 2–3x per week / Weekly / Monthly / Once / Never

2. Did the game encourage you to express your appreciation to a colleague?
   Yes / No / Unsure

*Optional: Explain:

---

3. Did the game encourage you to do something for your mental and/or intellectual health?
   Yes / No / Unsure

*Optional: Explain:

---

4. Did the game encourage you to do something for your physical health?
   Yes / No / Unsure

*Optional: Explain:

---

5. Did the game encourage you socialize with colleagues?
   Yes / No / Unsure

*Optional: Explain:

---

6. What did you like most about the game?

---

7. What did you like least about the game?

JMLA Journal of the Medical Library Association

SC
Supplemental content to
J Med Libr Assoc. Oct;108(4):dx.doi.org/10.5195/jmla.2020.742
www.jmla.mlanet.org
© Casucci, Locke, Henson, Qeadan 2020

8. Do you want to play again?

Yes / No / Not sure

9. Gender:

---

10. Age:
